# A Multi-Year Aerosol Characterization for the Greater Tehran Area Using Satellite, Surface, and Modeling Data

**DOI:** 10.3390/atmos5020178

**Published:** 2014-04-04

**Authors:** Ewan Crosbie, Armin Sorooshian, Negar Abolhassani Monfared, Taylor Shingler, Omid Esmaili

**Affiliations:** 1 Department of Atmospheric Sciences, University of Arizona, Tucson, AZ 85721, USA; 2 Department of Chemical and Environmental Engineering, University of Arizona, Tucson, AZ 85721, USA; 3 Department of Civil & Environmental Engineering, University of California, Irvine, Irvine, CA 92697, USA

**Keywords:** aerosol, Iran, MODIS, MISR, wet scavenging, visibility

## Abstract

This study reports a multi-year (2000–2009) aerosol characterization for metropolitan Tehran and surrounding areas using multiple datasets (Moderate Resolution Imaging Spectroradiometer (MODIS), Multi-angle Imaging Spectroradiometer (MISR), Total Ozone Mapping Spectrometer (TOMS), Goddard Ozone Chemistry Aerosol Radiation and Transport (GOCART), and surface and upper air data from local stations). Monthly trends in aerosol characteristics are examined in the context of the local meteorology, regional and local emission sources, and air mass back-trajectory data. Dust strongly affects the region during the late spring and summer months (May–August) when aerosol optical depth (AOD) is at its peak and precipitation accumulation is at a minimum. In addition, the peak AOD that occurs in July is further enhanced by a substantial number of seasonal wildfires in upwind regions. Conversely, AOD is at a minimum during winter; however, reduced mixing heights and a stagnant lower atmosphere trap local aerosol emissions near the surface and lead to significant reductions in visibility within Tehran. The unique meteorology and topographic setting makes wintertime visibility and surface aerosol concentrations particularly sensitive to local anthropogenic sources and is evident in the noteworthy improvement in visibility observed on weekends. Scavenging of aerosol due to precipitation is evident during the winter when aconsistent increase in surface visibility and concurrent decrease in AOD is observed in the days after rain compared with the days immediately before rain.

## 1. Introduction

Aerosol particles impact the planet’s energy balance, the hydrological cycle, atmospheric visibility, and public health. The relative strength of particles in imparting these effects depends largely on their abundance and physicochemical properties, which are governed by emission sources, transport, and meteorology. Particulate matter has been extensively monitored in several major urban centers of the world such as Beijing, Mexico City, and Los Angeles, but one area that has received disproportionately less attention in terms of examining the nature and character of aerosol is in the arid Middle East. This area is of importance with regard to air pollution owing to the large population, unfavorable topography for ventilation, and substantial sources of natural and anthropogenic emissions. In addition, this region is an important aerosol source for downwind receptors such as India, Pakistan and Afghanistan [1–3]; however, the relative contribution of Iranian sources to pollution in these receptor sites is unknown.

**Figure 1:**
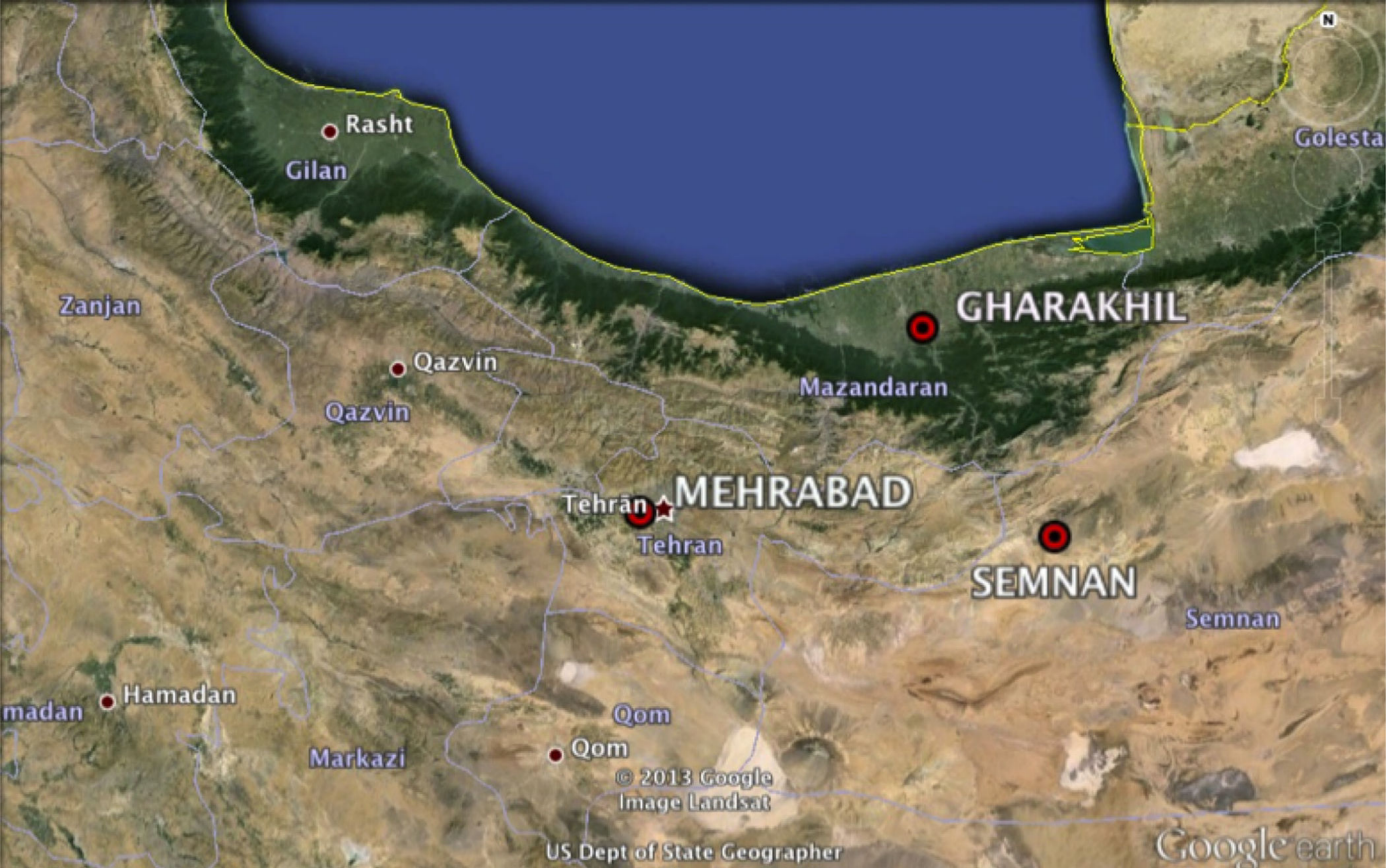
Geographic locations of the ground-based meteorological monitoring stations.

The capital city of Iran is Tehran (Figure 1) and the metropolitan area covers an area over 2300 square kilometers in the northern part of the country. The population of the Tehran metropolitan area has grown from 11.3 million in 2006 to 12.2 million in 2011 (United Nations Population Fund; http://iran.unfpa.org/). Air pollution problems are exacerbated in this major metropolitan area due to surrounding mountains, which extend to over 5000 m and inhibit ventilation of pollutants, especially during wintertime. The Alborz Mountains provide a more prominent meteorological barrier to many other urban regions, which suffer topographical blockage in the presence of a subsidence inversion (e.g., San Gabriel and San Bernardino Mountains/Los Angeles, Wasach Mountains/Salt Lake City and Cordillera Neovolcánica/Mexico City). The growing population and extensive anthropogenic emissions result in major air quality issues and uncertain effects on the region’s microclimate and hydrological cycle. Visibility data from the last 50 years indicate a long-term trend in visibility reduction and suggest that worsening air quality is attributable to emissions rather than meteorological factors [4]. Vehicular emissions are of particular concern in the region owing to more than two million vehicles, many of which are more than two decades old [5]. Dust has a substantial impact on the region owing to internal sources [6–8], including numerous dry lakes (e.g., Hamun-e Jaz Murian, Hamun-i-Mashkel, Daryacheh-ye Mamak) and the larger Dasht-e Kavir Desert, and external sources due to its location near the Arabian Peninsula to the south and the Euphrates and Tigris Basins to the west [8–11]. PM_10_ concentrations in parts of Iran can reach more than 5 mg·m^−3^, which consequently contribute to enhanced mortality [10]. It was reported that in the city of Zanjan, just to the northeast of Tehran, the dominant aerosol type was dust and that only 20% of all particles were smaller than 1 µm [12]. Dust has also been shown to be more abundant in parts of Iran during spring and summer, while motor vehicles are more influential during fall and winter and during weekdays [13].

Owing to limited surface measurements of particulate matter in the Tehran metropolitan area, satellite remote sensing data are highly valuable in examining spatiotemporal patterns of air pollution in this area [14]. A number of aerosol climatology studies have been conducted in different global regions (e.g., Greece, Israel, Pakistan, China, Egypt, southwestern United States, South Africa, India) to critically examine temporal aerosol characteristics [2,15–23], including the relationship with air mass source origins and seasonality [24,25]. Concerted efforts to combine satellite data with available surface measurements, air mass back-trajectory data, and chemical transport model results do not exist for the Tehran area and its surroundings, a fact that motivates the current study.

The goal of this work is to report a multi-year (2000–2009) aerosol characterization for metropolitan Tehran and surrounding areas with an aim to extend upon previous studies examining air pollution characteristics in Iran. This work addresses the following questions: (i) what are monthly trends in aerosol-related parameters and others that potentially influence them such as meteorology and air mass source regions? (ii) What is the role of precipitation in modulating aerosol-related parameters? And (iii) can the datasets provide any indication of the weekly aerosol cycles and the relative strength of dust *versus* other aerosol types? In addressing these questions, we will determine the degree of correspondence between satellite data, chemical transport modeling, and surface measurements of visibility.

## 2. Data and Methods

Direct surface measurements of aerosol are not publicly available for Tehran during the study period. In light of this limitation this study leverages a combination of remotely-sensed data, local meteorological observations and model data.

### 2.1. Remotely-Sensed Aerosol Data

A summary of spatial areas and time durations of satellite data collection, downloaded from the NASA GES DISC Giovanni online data system, is reported in Table 1. Daily Level 3 data were obtained from the Moderate Resolution Imaging Spectroradiometer (MODIS) Version 5.1 [26], Total Ozone Mapping Spectrometer (TOMS) Version 8 [27], Ozone Monitoring Instrument (OMI) Version 3 [28,29], and the Multi-angle Imaging Spectroradiometer (MISR) Version 31 [30,31]. Data from MODIS include the daily 1° × 1° gridded Deep Blue AOD (0.55 µm) [32]. The Deep Blue algorithm is appropriate for desert land surfaces as it is sensitive to particles over bright surfaces [33], with AODs being within 20%–30% of those measured by sun photometers [33]. The Deep Blue algorithm has also been extensively tested for purposes of aerosol data assimilation over North Africa and Southwest Asia [34], and furthermore, MODIS Deep Blue AOD data were found to compare well with sun-photometer measurements at Zanjan, Iran between 2006 and 2008 during dust events [12]. MISR AOD data (0.555 µm) are used between 2000 and 2009 at a resolution of 0.5° × 0.5° [35]. Ultraviolet Aerosol Index (UV AI) data are used from TOMS (1° × 1.25°) and OMI (1° × 1°) for a representation of the relative abundance of absorbing aerosols [36], including dust and smoke. A minimum UV AI threshold of 0.5 is applied in this study to account for instrumental uncertainties [37]. To account for cloud contamination over land, remotely sensed aerosol data are only used when the MODIS cloud fraction is less than 70% [38]. The aim of this work is to use these datasets to examine patterns in retrieved parameters and for inter-comparison with other observational and model data.

### 2.2. Meteorology

Meteorological data were obtained from the National Climatic Data Center (NCDC) for three surface stations in northern Iran (Figure 1, Table 1): Mehrabad International Airport (35.68°N, 51.32°E), Gharakhil (36.45°N, 52.82°E), and Semnan (35.55°N, 53.38°E). Data include dry bulb temperature, dew point temperature, visibility, wind speed, and precipitation. Relative humidity (RH) was subsequently calculated from the surface dry bulb temperature and dew point temperature. The three sites were selected for their respective microclimates in the context of the local topography and proximity to urbanization. The sites are summarized as follows: (i) Mehrabad is located at Tehran’s main international airport within the city metropolitan area in the southwest quadrant; (ii) Gharakhil is in the Mazandaran province, which is situated on the north slopes of the Alborz mountain range near the southern coast of the Caspian Sea; and (iii) Semnan is an industrial area east of Tehran. They provide a range of conditions especially since Gharakhil is on the other side of a major topographical feature to contrast to Mehrabad, which is the most urban-impacted site within the Tehran metropolitan area.

In addition to surface data, radiosonde data were obtained from Mehrabad International Airport. Data were analyzed at twice daily intervals (00:00 UTC and 12:00 UTC) between 1980 and 2012. Archived data from the Modern-Era Retrospective Analysis for Research and Applications (MERRA) from NASA Goddard GMAO [39] provided additional data, specifically integrated column water vapor (CWV) and planetary boundary layer height (PBLH). Model grid spacing is 0.5° latitude and 0.67° longitude and data at hourly time increments were used in order to understand diurnal and seasonal cycles.

### 2.3. Air Mass Back-Trajectory Data

To determine air mass source origins impacting Tehran, five-day back-trajectories were computed using the NOAA HYSPLIT (Hybrid Single-Particle Lagrangian Integrated Trajectory) model [40], which was run using the NCAR/NCEP reanalysis data with the isentropic vertical velocity method. Six-hourly trajectories from 2000 to 2009 were obtained ending at Tehran (35.70°N, 51.42°E) at 500, 1000, and 3000 m above the surface. The HYSPLIT data were used to construct seasonal (DJF, MAM, JJA, SON) trajectory frequency maps for the 2000–2009 period, which present the most frequent transport pathways of air ending in Tehran. Trajectories are also classified by source region denoted by regions A–E: A = desert region southwest of Tehran; B = Europe; C = Siberia/Russia; D = countries which are east of Iran; E = representation of local sources in and around Tehran.

### 2.4. Satellite Fire Data

Fire data from MODIS Fire Information for Resource Management System (FIRMS) were collected from 2000 to 2009 over a domain spanning 20°N to 58°N and 30°E to 73°E. Fire Radiative Power (FRP) is related to the burn intensity of the fire pixel and is provided for each identified fire per overpass. For purposes of estimating aerosol emissions related to biomass burning the FRP is integrated over the area within each 0.5° × 0.5° grid box within the entire domain. Data are presented as a time average of the area integrated FRP for each season.

### 2.5. Goddard Ozone Chemistry Aerosol Radiation and Transport (GOCART) model

Daily predictions of the total optical depth (0.55 µm) associated with various aerosol components (sea salt, sulfate, dust, organic carbon, and black carbon) were provided by the GOCART model [41] at a resolution of 2° × 2.5° from 2000 to 2007.

## 3. Results

### 3.1. Local Meteorology

The local meteorology is examined using surface station data at Tehran (Mehrabad) and compared with data from the nearby stations at Gharakhil and Semnan to understand the impact of local microclimatic variability within the context of the region. In addition, twice daily long-term radiosonde data from Mehrabad are used to provide a characterization of the vertical profile of the atmosphere (dry-bulb and dew-point temperature) throughout the annual cycle. Global reanalysis data from MERRA are used in conjunction with the radiosonde soundings to determine the monthly trends in mixing layer heights, which have important consequences for the seasonal changes in the vertical transport and mixing of aerosols.

**Figure 2:**
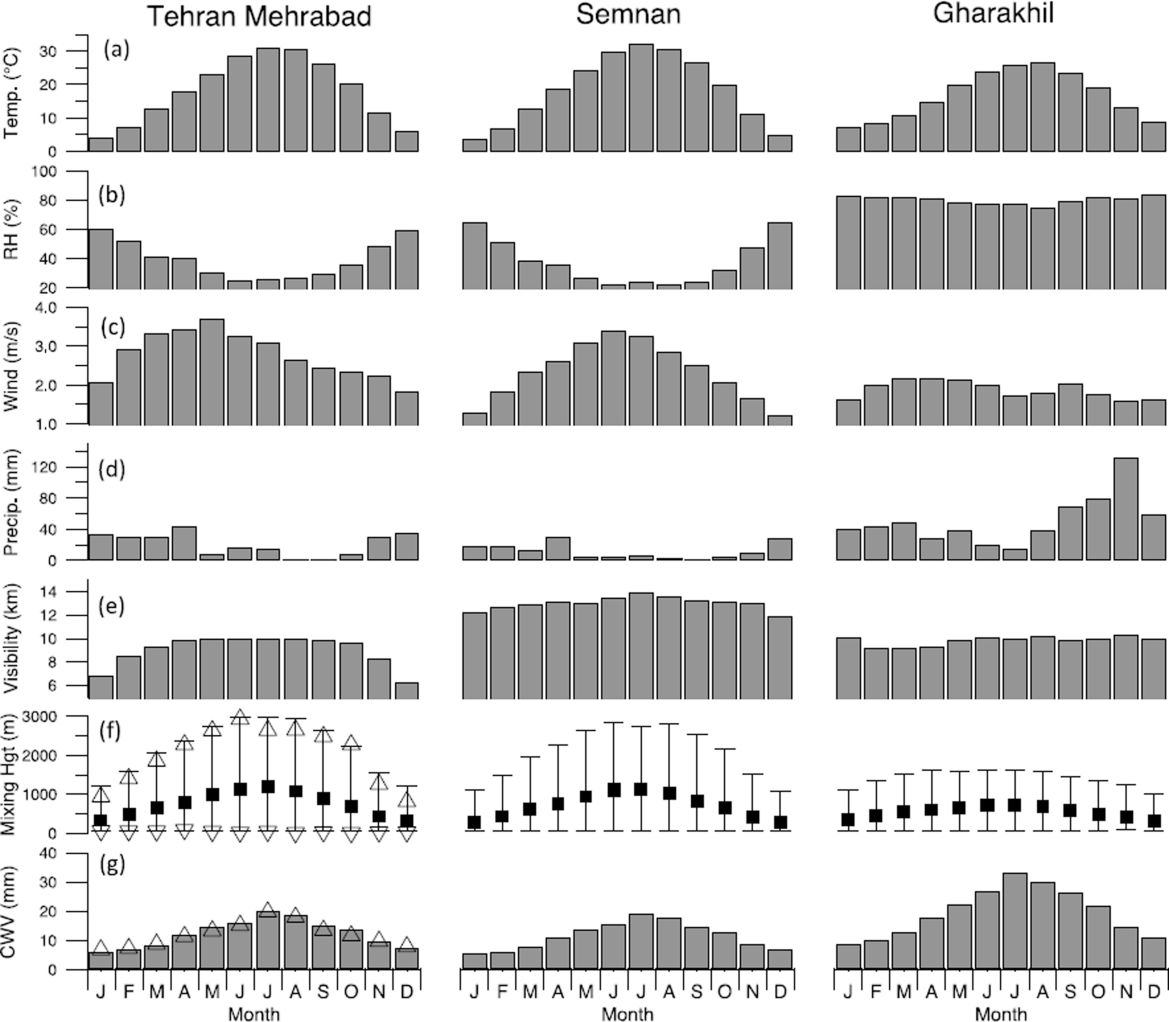
Monthly summary of surface meteorological data at three sites near Tehran (see Figure 1 for locations) between 2000 and 2009 for (**a**) dry bulb temperature (T), (**b**) relative humidity (RH), (**c**) wind speed, (**d**) accumulated precipitation, and (**e**) visibility. Monthly summary of upper air data for the same sites: (**f**) mixed layer height derived from Mehrabad radiosonde data (1980–2012; 00Z and 12Z soundings shown as triangle markers) and MERRA reanalysis data (2000–2009; square markers represent daily mean and whiskers represent average daily range) at grid points near Tehran (35.50°N, 51.33°E), Semnan (35.50°N, 53.33°E) and Gharakhil (36.50°N, 52.67°E); (**g**) Same as (f) except for average total column water vapor (CWV).

A summary of the monthly average surface station data at Mehrabad, Gharakhil and Semnan is shown in Figure 2a–e. The region is characterized by a semi-arid climate, with hot and dry summers, cold winters, and mild conditions in the spring and fall. The annual cycle of ambient surface temperature is expectedly very similar at Mehrabad and Semnan ranging from as low as 3.7 °C in January to 32.2 °C in July and with monthly averages deviating by approximately 1 °C between the sites. Gharakhil has a slightly lower seasonal variability, because of its proximity to the Caspian Sea, with temperatures ranging from 7.2 °C in January to 26.6 °C in August. At Mehrabad, wind speeds at the surface are lowest in the winter months and peak in the spring and early summer. Semnan has comparable wind speeds during the summer but in the other seasons the wind speed is lower. Gharakhil has lower wind speeds overall with minimal seasonal variability. Precipitation accumulation at Mehrabad and Semnan is generally low throughout the year with minimal rainfall from May to October. April is the wettest month for both stations with 45 mm at Mehrabad and 30 mm at Semnan. Both of these stations receive 80% of their annual precipitation between November and April. Gharakhil experiences a markedly different precipitation regime with comparable totals during the early part of the year followed by a large upswing starting late summer and extending through the fall to early winter with precipitation peaking at 132 mm in November. The surface RH at Mehrabad and Semnan follows a very similar seasonal pattern with the driest conditions found during the summer months because of the high ambient temperatures. Gharakhil is humid throughout the year with little seasonal variability.

Monthly patterns in mixing layer height (Figure 2f) and CWV (Figure 2g) were calculated using the radiosonde data at Mehrabad in conjunction with the values derived from the MERRA dataset at grid points near Tehran (35.50°N, 51.33°E), Semnan (35.50°N, 53.33°E) and Gharakhil (36.50°N, 52.67°E). The mixing layer height was calculated from the radiosonde data using a threshold where the gradient in the potential temperature exceeded 0.02 K/mb. There is a noticeable increase in the daily maximum mixing layer height during the summer compared with the winter and increased mixing layer heights are found on the south side of the mountains (Tehran and Semnan) consistent with expectations for a semi-arid sub-tropical climate. The diurnal cycle of the mixing layer (not shown) shows the characteristic maximum in the afternoon driven by solar heating at the surface and overnight surface inversion typical of desert environments. Gharakhil shows a similar diurnal pattern with slightly less variation and has the signature of the influence of afternoon sea breezes. CWV (Figure 2g) is at a maximum during the summer at all locations, consistent with higher temperatures and hence higher saturation vapor pressures. The peak monthly values occur in July for all three locations reaching 20 mm at Tehran, 19 mm at Semnan, and 33 mm at Gharakhil.

The average visibility (Figure 2e) at Semnan is highest in July at 13.9 km with a moderate reduction observed during the winter, reaching a minimum of 11.9 km in December. Mehrabad also follows the same annual pattern; however, the visibilities are systematically lower for all months with a considerable reduction during the winter with minima of 6.3 km in December and 6.8 km in January. In both cases, local aerosol emissions are trapped within the surface layer leading to higher surface concentrations and lower visibility in the winter. The contrast between the two stations highlights the magnitude of the local aerosol sources in Tehran, which strongly affect the Mehrabad data but have a smaller effect on Semnan. In complete contrast, the annual visibility profile at Gharakhil follows a different pattern with minimal variability throughout the year (range ~1.2 km *versus* ~3.8 km atMehrabad). This is likely due to the different meteorological conditions north of the Alborz Mountains where reduced visibility may be associated with phenomena other than an increase in aerosol concentration, such as fog or rain.

### 3.2. Air Mass Source Origin

**Figure 3:**
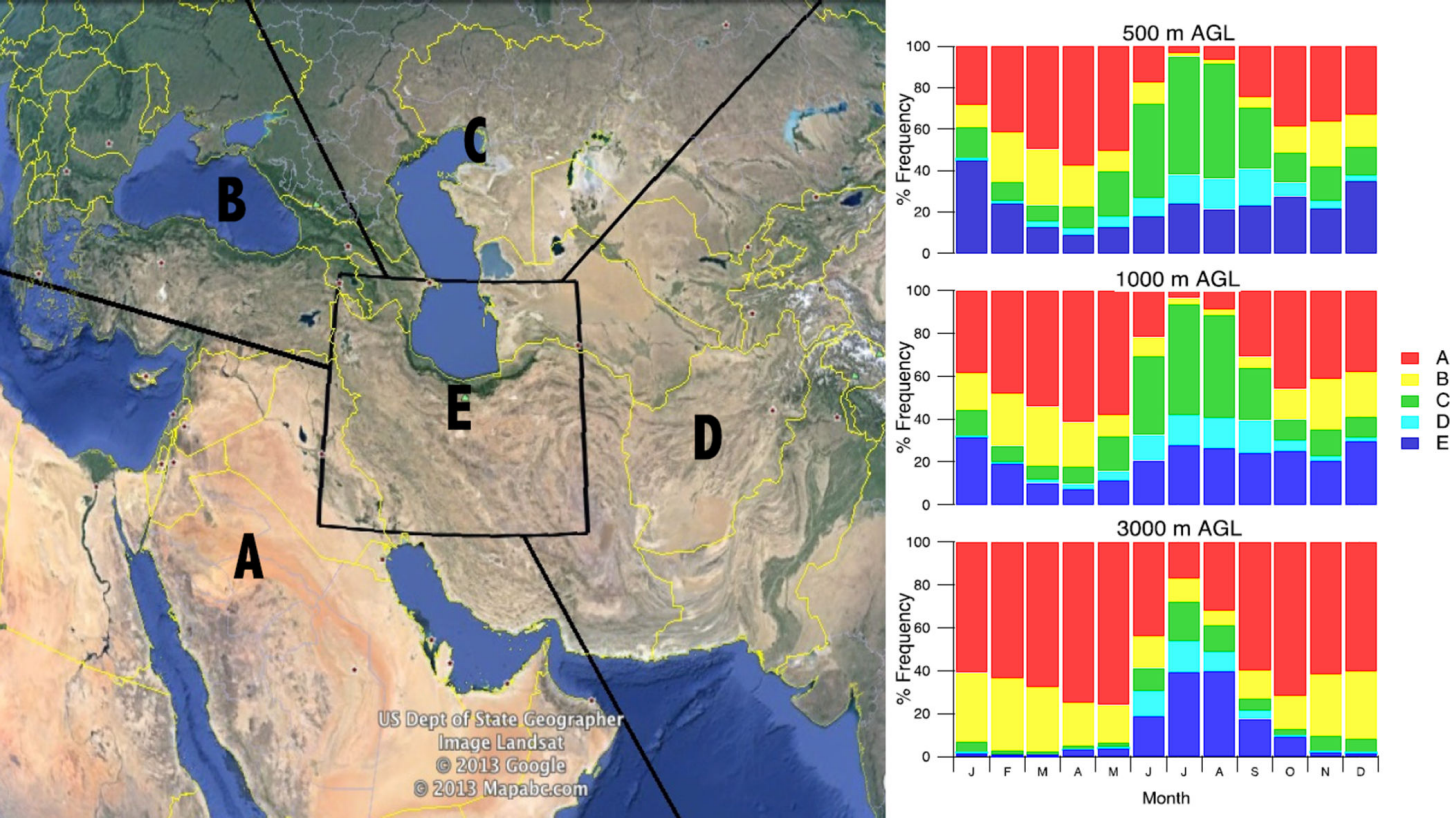
Monthly pattern in air mass source region as determined by analysis of daily Hybrid Single-Particle Lagrangian Integrated Trajectory (HYSPLIT) data between 2000 and 2009. Back-trajectories are classified by time spent in each region. The region totals are shown, by month, for end points at 500 m, 1000 m and 3000 m above ground level (AGL) (**Right**).

**Figure 4:**
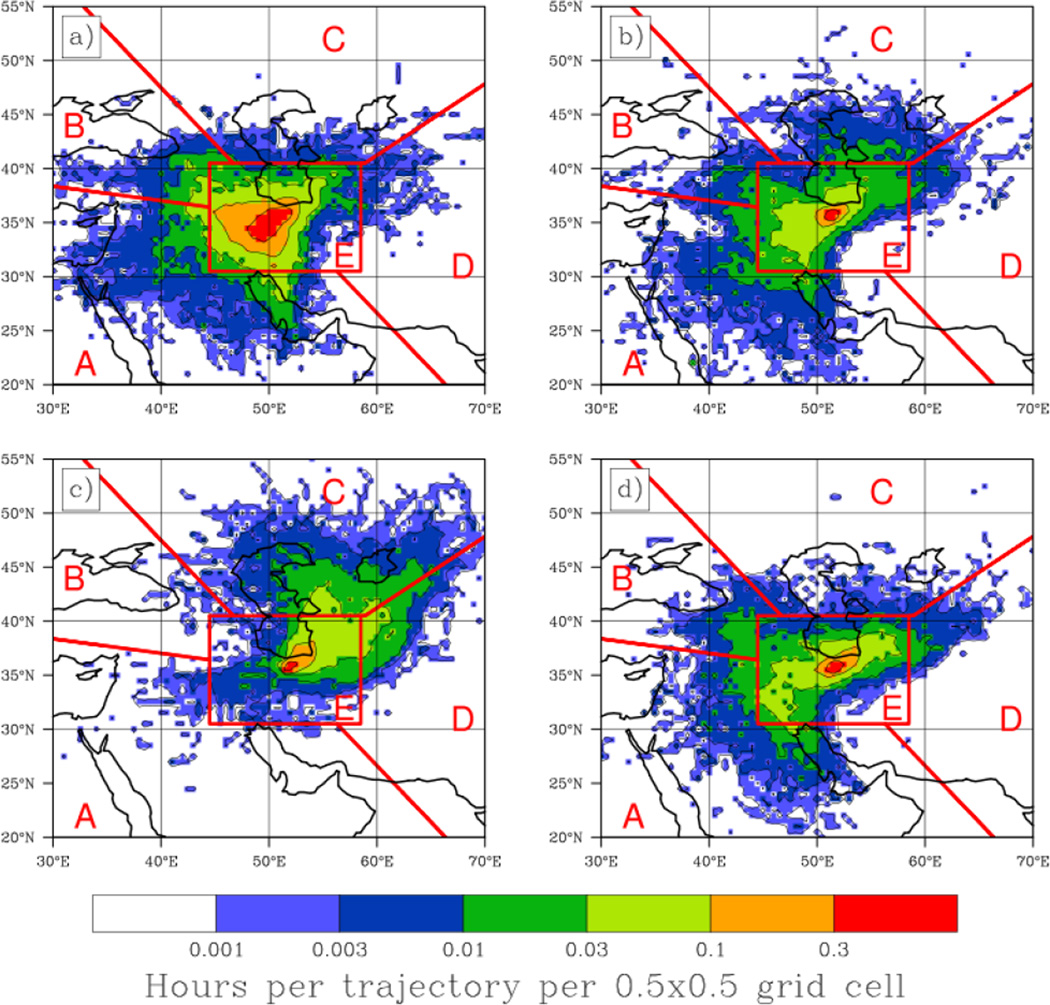
Decadal (2000–2009) summary of seasonal HYSPLIT five-day back-trajectory frequencies, ending at 500m above Tehran (35.70°N, 51.42°E) for (**a**) winter (DJF), (**b**) spring (MAM), (**c**) summer (JJA), and (**d**) fall (SON). Frequency is defined as the number of trajectory-hours spent in each 0.5° × 0.5° grid box divided by the total number of trajectories analyzed. Source regions, as illustrated in Figure 3, are overlaid.

An important factor governing aerosol characteristics in the greater Tehran area is the seasonal air mass transport pathways ending at this location. Trajectories ending below 1000 m show qualitatively similar patterns with altitude (Figure 3) and during the majority of the year, the most dominant source region (defined in Section 2.3) is found to be in the desert regions to the west and southwest (Region A). In summer the circulation pattern of the region is significantly different and trajectories from Region C prevail at low levels. Low-level trajectories in winter (DJF) included a significant contribution from Region E (34%), which is indicative of stagnant low-level air. While HYSPLIT may not resolve these features entirely, the stagnation at the surface would be further enhanced by shallow mixing heights with stable air aloft which would trap air below the mountain tops. The least important source regions were from Regions B (northwest) and D (east). The importance of trajectories originating from the dust-rich region between the west and south of Tehran increased for the upper levels. At an ending altitude of 3000 m AGL, the air mass origins were in Region A for 57% of trajectories annually and reached a peak fraction during the spring (MAM) of 73%. For trajectories ending below 1000 m, there was no significant change in the attribution of source region when only surface influenced (<500 m) fractions of the trajectory were considered (not shown). Low-level (ending altitude 500 m AGL) trajectory density is presented as an average residence time (hours per trajectory) within each cell in a 0.5° × 0.5° grid to supplement the apportionment of source regions (Figure 4). Consistent with Figure 3, all seasons except summer (Figure 4c) exhibit similar trajectory maps with air mass source regions in a quadrant from the south through to the west and a secondary source region to the northeast, although many of these trajectories were likely classified as Region E. The summer source region is predominantly to the north and northeast, which accounts for the abundance of Region C trajectories during these months.

### 3.3. Regional Fire Patterns

**Figure 5:**
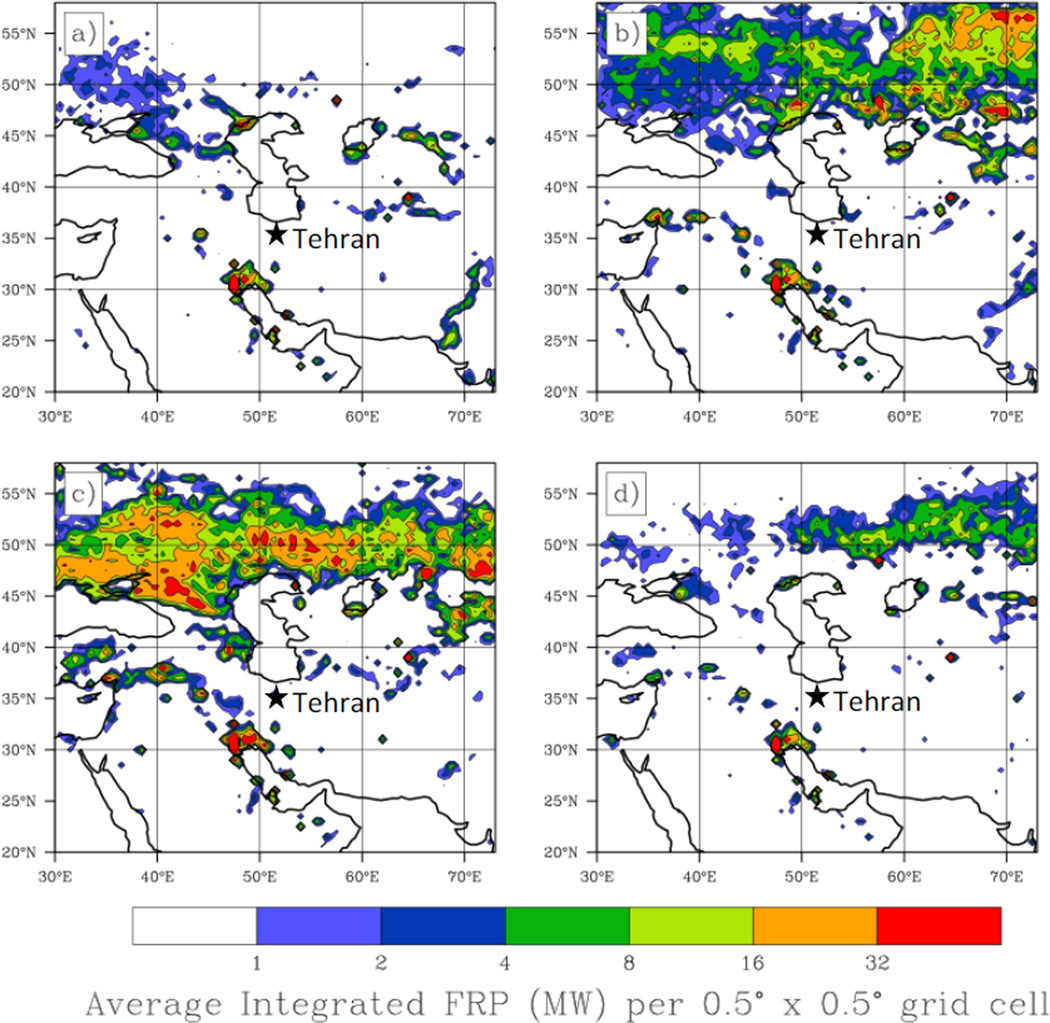
Seasonal patterns in Fire Radiative Power (FRP) derived from Moderate Resolution Imaging Spectroradiometer (MODIS) Fire Information for Resource Management System (FIRMS) data from 2000 to 2009. FRP is shown as integrated seasonal average power (megawatts per 0.5° × 0.5° grid box) for (**a**) winter (DJF), (**b**) spring (MAM), (**c**) summer (JJA), and (**d**) fall (SON).

Using the FIRMS data from 2000 to 2009, a FRP climatology has been developed for four seasons for a region spanning eastern Europe, central Asia and the Middle East (Figure 5). FRP across the region increases in spring and summer. In particular, the region to the north of the Caucasus Mountains extending into Ukraine, southwest Russia and further northeast into Kazakhstan experiences a substantial number of fires during the summer. The fires in Ukraine and southwest Russia are predominantly associated with agricultural burning. During spring, there is a maximum in fire density shifted farther east into Kazakhstan. The number of fires during fall and winter is significantly lower than other seasons and is likely to have little impact on aerosol concentrations in the study region.

### 3.4. Remotely-Sensed Aerosol Data

**Figure 6:**
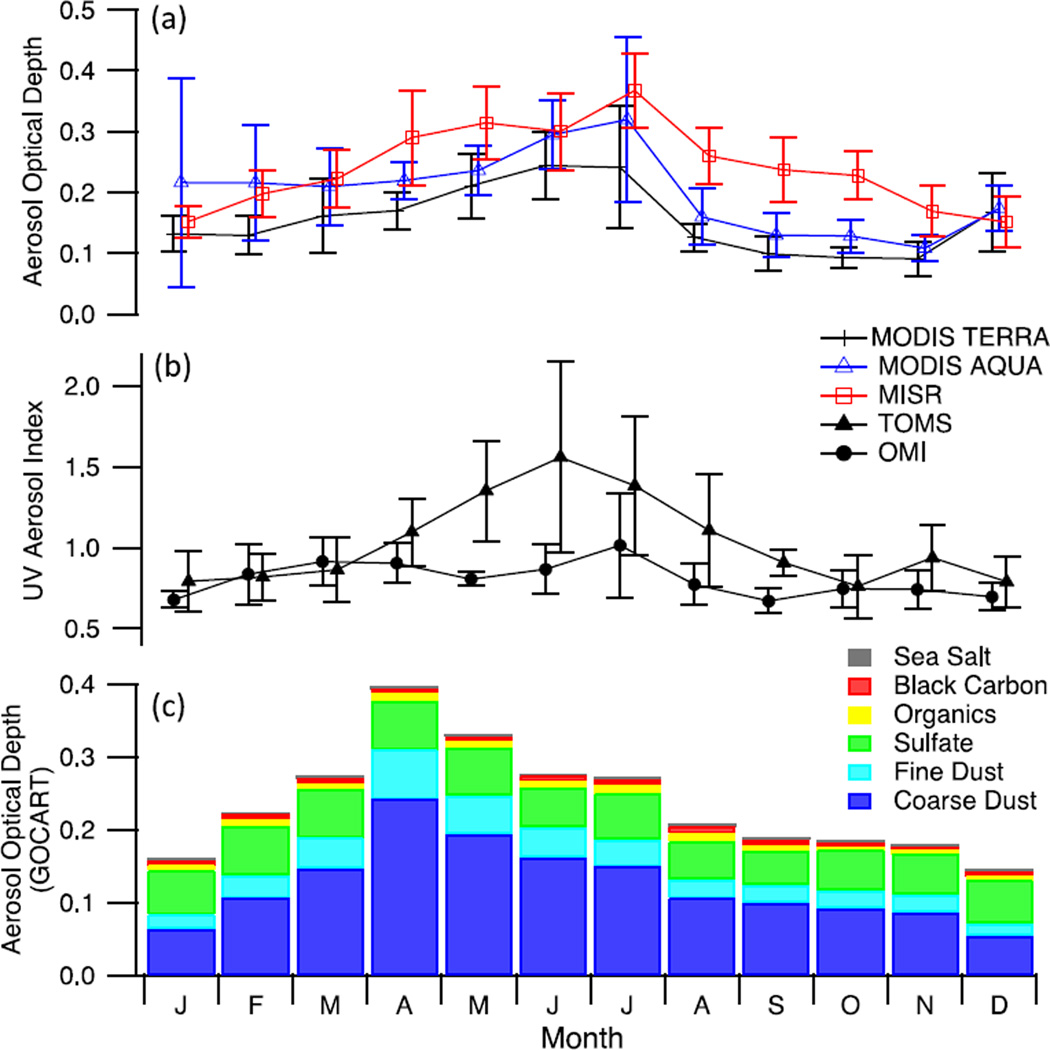
Monthly summary of remotely-sensed and model data for the greater Tehran area (see Table 1) for different satellite products (a and b): (**a**) aerosol optical depth (AOD) from MODIS Deep Blue (Terra and Aqua) and Multi-angle Imaging Spectroradiometer (MISR); (**b**) Total Ozone Mapping Spectrometer (TOMS) and Ozone Monitoring Instrument (OMI) ultraviolet aerosol index; (**c**) monthly summary of fractional AOD from Goddard Ozone Chemistry Aerosol Radiation and Transport (GOCART).

MODIS Deep Blue (Terra and Aqua) and MISR all show that AOD is largest between April and August (Figure 6a). Although not presented quantitatively, the MODIS Angstrom Exponent (AE) monthly averages were also considered as a qualitative method of assessing coarse mode *versus* fine mode aerosol. Lower AE values are generally found in the spring and summer months, which suggests a shift towards coarser aerosol such as dust. This point is further supported by Figure 6c where it is shown that the highest UV AI values are observed in the spring and summer (May–July), especially for the TOMS sensor. However, there are two possible mechanisms for the significant upswing of UV AI levels during the summer: an increase in the abundance of absorbing aerosol (*i.e.*, dust and smoke) or a change in the column distribution of the absorbing aerosol. While an increase in overall dust concentration is the likely contributor to the increase in UV AI, it may also be driven by an increase in elevated dust and/or smoke layers. Some caution must be employed when considering the mean AOD and UVAI values during the winter, since during this time of year there were fewer data points available, because of cloud contamination. To qualitatively assess the significance of the seasonal cycle we evaluate monthly mean values against the standard deviation of interannual variability(shown as error bars on Figure 6). Winter AOD is generally more variable than summer; however, in summer the range of variability is amplified, particularly for the TOMS data, in part due to extreme dust events, which occur in some years and not in others.

### 3.5. GOCART

Data from GOCART simulations were used to quantify the relative importance of different aerosol constituents. The monthly average fractional AOD for fine and coarse dust, black carbon, organics, sulfate, and sea salt are shown in Figure 6d. In examining these data, we focus on the relative fraction of the constituents and their seasonal trends instead of considering the absolute values to reduce sensitivity to model limitations.

Throughout the entire annual cycle, dust optical depth (which includes coarse and fine dust), accounts for the largest fraction of the total optical depth at 68% of the average annual aerosol with highest levels during April. Sulfate contributes the next highest fraction with an annual average of 25% with little variability through the year. Black carbon and organics are responsible for a relatively small fraction of the total AOD at 2.8% and 3.6%, respectively; however, the peak occurs during July and August and is suggestive of a biomass-burning source due to wildfires mainly in Ukraine, Russia and Kazakhstan. The peak AOD from GOCART occurs during spring, which is consistent with the influx of regional and local dust; however, the model may be under predicting the role of black carbon and organics associated with biomass burning due to uncertainties in the emissions inventory. In addition, GOCART does not include gas phase chemistry and hence cannot suitably model secondary production of aerosol that is not approximated at the source. Sea salt is also a very low impact contributor to the total AOD (approximately 0.6% annual average) with the peak occurring during winter and early spring where upper air trajectories are from the west and southwest. The HYSPLIT trajectories suggest that sea salt aerosol sources include the Persian Gulf and the Mediterranean Sea and perhaps the Caspian Sea, although its salinity is far lower. The high terrain and lack of local sea salt sources suggests that marine air intrusions do not affect the lower troposphere and this is confirmed by the contrast in meteorology at Mehrabad and Semnan as compared to Gharakhil (see Section 3.1) and justifies the lack of sea salt aerosol.

## 4. Discussion

### 4.1. Seasonal Climatology

Many of the patterns found in the surface data conform to the expected seasonal variability, which is characteristic of a sub-tropical desert environment such as temperature, humidity, and visibility. The observed meteorology at Mehrabad, Gharakhil and Semnan can be explained by the influence of local topography, land surface, urbanization, and large-scale atmospheric circulation pattern of the region. Further analysis of the data suggests mechanisms for variability in aerosol quantified using satellite-derived AOD and surface visibility. Since satellite AOD was available only on days with low cloud fraction, there was a potential sampling bias associated with the comparison of seasonal visibility and AOD cycles. However, the difference between the visibility statistics derived on days when satellite data were available and the entire dataset was found to be negligible, and so this bias was not relevant to this study.

Using the visibility data at Mehrabad as a proxy for surface-layer aerosol concentration reveals that the reduced visibility during winter is aligned well with recurring reports of hazardous air quality within the city being more prevalent during this season. MODIS Deep Blue and MISR data indicate that AOD is lowest during winter. For this to happen the distribution of aerosol through the column is more weighted towards the near-surface layer. The local meteorology supports this conclusion, since average mixing layer heights are far lower in winter and stable air above the mixing layer traps air below the mountains causing a high incidence of stagnant air at the surface, which is infrequently ventilated. In contrast, the summer exhibits a maximum in the satellite-derived AOD and the highest visibilities at Mehrabad. Mixing heights are highest mainly due to the high incident solar radiation, which vigorously mixes aerosol in the lower troposphere and helps to relieve the accumulation of aerosol near the surface. The AOD is highest during this season, which suggests a higher columnar aerosol concentration, and is likely attributed to dust transport in the mid- to upper-troposphere from source regions in the deserts to the west of Iran (Arabia, North Africa, and the Levant), although it is unclear exactly which dust source regions are most influential for Tehran. While data for AOD and perhaps TOMS and OMI UV AI indicate that dust is most important during spring and summer, the trajectory analysis only supports the argument for regional dust transport during spring. Nonetheless, there is evidence from individual cases that regional dust transport can contribute to extreme events during the summer. In addition, there is an abundance of local sources of dust within Iran, in relative proximity to Tehran, and these sources may be most impactful during summer due to higher surface wind speeds and potentially lower soil moisture. One possible method for isolating local and regional dust sources is the ratio of PM_2.5_ to PM_10_, with lower ratios suggestive of greater influence from local dust sources [42]; that study suggested 0.35 as a threshold value, above which data are contaminated by non-local dust sources. Results from [10] for PM_2.5_ and PM_10_ concentrations measured at a site in western Iran during 2010, suggest a range of PM_2.5_:PM_10_ between 0.18 and 0.32 (based on ratios of monthly-averaged values) indicating that local dust sources make a significant contribution.

Another mechanism for the enhancement of satellite-derived AOD during the summer may be the swelling of aerosol due to uptake of water vapor (*i.e.*, hygroscopic growth). Higher CWV in the summer (Figure 2g) supports the occurrence of hygroscopic growth, and even though surface relative humidity values are suppressed (Figure 2b), the relative humidity in the upper parts of the (deep) mixing layer (not shown) is sufficient for significant water vapor uptake. Later in the summer, upper air trajectories imply that air mass origins from the north are prevalent, which may indicate a contribution from biomass burning sources due to smoke from wildfires in Ukraine and Russia. Although the magnitudes are too small to be of major significance, if taken in a relative sense, GOCART generally supports this with an increase in black carbon optical depth during the summer.

### 4.2. Precipitation

**Figure 7:**
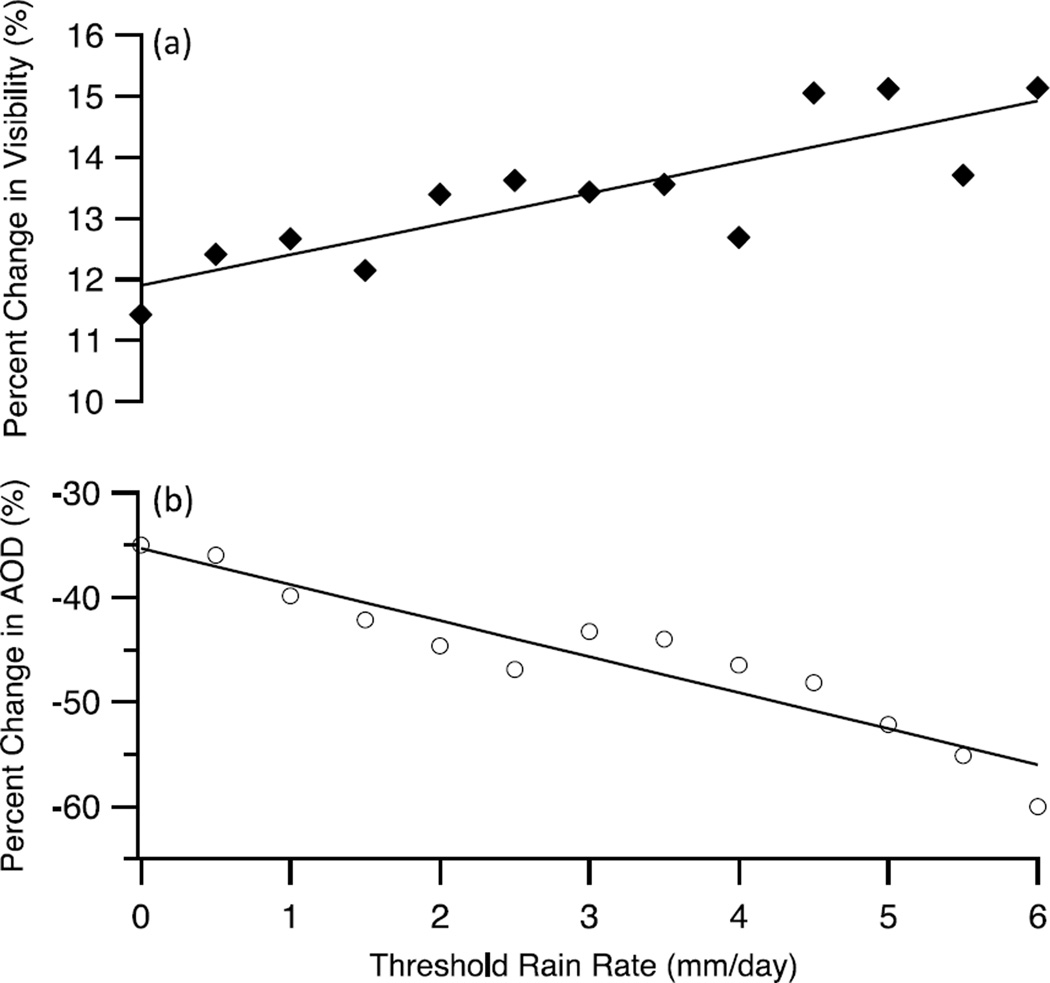
Change in (**a**) visibility and (**b**) satellite-derived AOD immediately before and after rain days. The plots show the composite average difference between the mean visibility/AOD during the two days after rain and the two days before rain. The composite is taken for rain events, which exceed the given threshold daily rainfall rate and is presented as a percentage with respect to the mean visibility/AOD before the rain.

The discussion above suggests that the combination of local and regional aerosol sources is stronger during the summer, however, the mechanism for the removal of aerosol can be equally as important. An essential mechanism for modulation of aerosol loading is the scavenging of aerosol by precipitation. Summer (JJA) rain in Tehran is rare, and the average interval between rain events at Mehrabad during 2000–2009 is 23.5 days compared with 4.5 days for winter (DJF), 5.0 days for spring (MAM), and 8.2 days for fall (SON). To understand the importance of this interaction for the climatology of Tehran, we investigate the difference in aerosol immediately before and after rainy days, which are defined as days with observed rainfall at Mehrabad. We focus only on the winter months, since this is the season with the most rain days and also is the most critical season in terms of aerosol effects on public health owing to a shallower mixing layer accumulating a higher concentration of pollutants near the surface. Figure 7a shows the composite average change in visibility at Mehrabad between the mean visibility during the two days before and two days after rainfall stratified by the severity of the rainfall event and Figure 7b shows the same comparison for AOD. Since the number of rain events is small and there is considerable loss of AOD data surrounding rain events due to cloud contamination, a “consolidated” AOD is generated using the three satellite products used in this study (MODIS Deep Blue (Terra and Aqua) and MISR). We take the available data from the three products, and for instances where more than one measurement exists, we take the (unweighted) mean. Consequently this alleviates the fact that each product is not available for the entire study period. There is a significant increase in visibility and concurrent reduction in AOD at all rain rates and furthermore, there is a general trend showing that the magnitude of the change in visibility and AOD increases as the severity of the rainfall increases. Overall, this finding suggests that rainfall events tend to have a beneficial impact on the extreme aerosol concentrations found in Tehran during the winter.

### 4.3. Trajectory Analysis of Extremes

**Figure 8:**
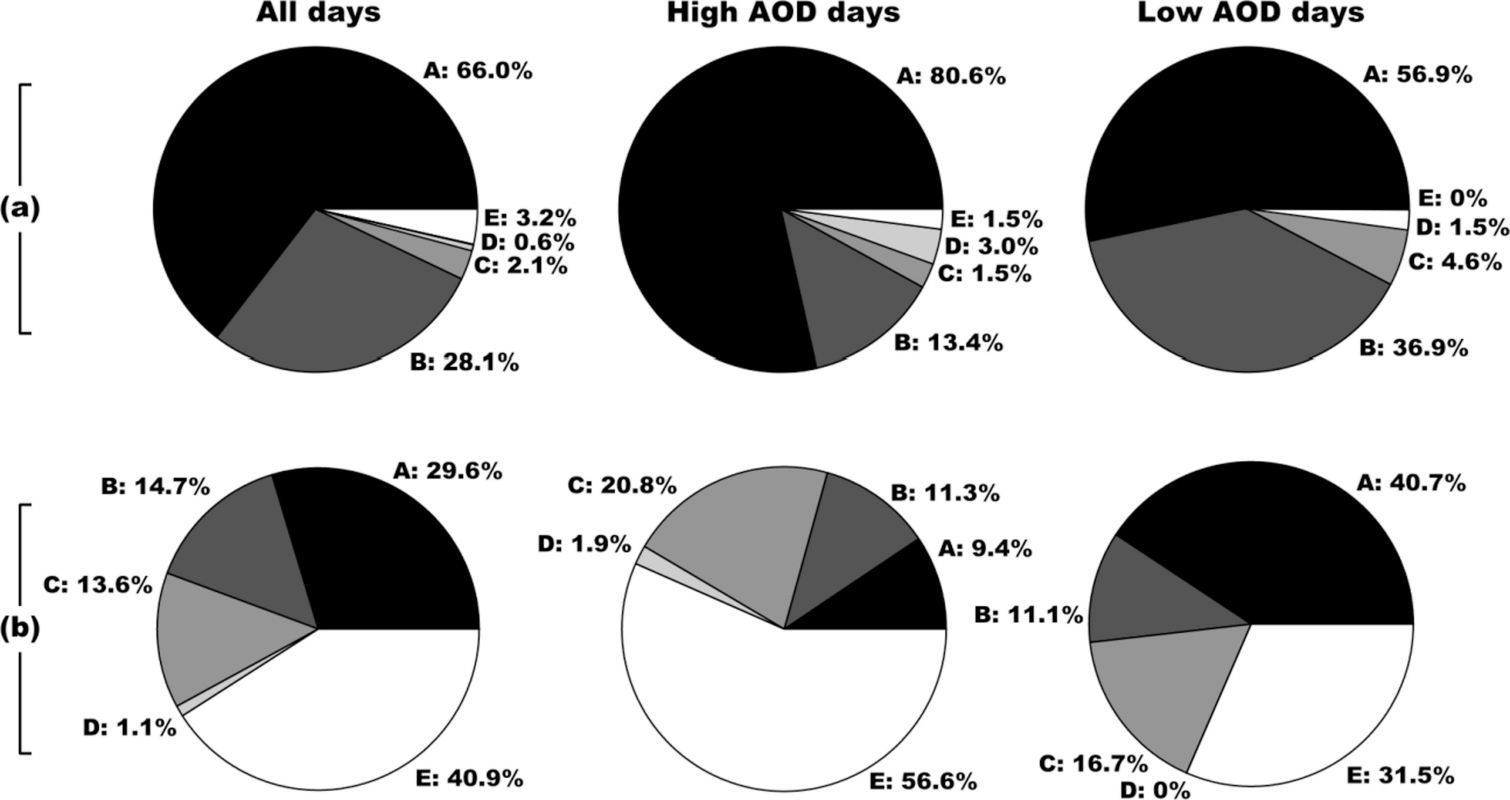
Air mass source origin for all days, high AOD days (>90th percentile) and low AOD days (<10th percentile) for (**a**) spring upper air trajectories (ending altitude of 3000 m AGL) and (**b**) winter low level (500 m AGL) trajectories. High and low AOD days were identified using the consolidated MODIS Deep Blue (Terra and Aqua) and MISR AOD data.

The spring season exhibits a significant increase in AOD, and it is of interest to investigate the mechanisms responsible for this. The effect of precipitation washout is certainly in favor of this trend since monthly-accumulated precipitation at Mehrabad decreases rapidly from April into May. Trajectory analysis shows that the prevailing upper air origins are the deserts to the west of Iran (Region A) during the winter and spring; however, there is a more preferential bias for Region A against Region B (Europe) during spring compared to winter. This result would indicate that there was the potential for increased long-range dust transport into Tehran and the surrounding areas during spring. The distribution of air mass origin during this season was further refined by considering a subset of trajectories corresponding to the extremes of the consolidated AOD data (see Section 4.2). The top and bottom 10% of observed daily AOD were analyzed to identify if there was a change in the distribution of upper air origins for “high” *versus* “low” AOD days (Figure 8a). It is clear that there is a higher-than-average fraction of back-trajectories that originate in the dust-rich Region A during high AOD days compared with low AOD days.

A similar analysis is also performed for surface trajectories during the winter and is also shown in Figure 8b. The main finding is that the “high” AOD days contain an abundance of stagnant trajectories (Region E) compared with low AOD days, which show a higher prevalence of trajectories from the west (Regions A and B). Whilst this result may appear to conflict with the postulation that regional dust transport from Region A at higher levels leads to an increase in AOD during spring and summer, the presence of westerly winds near the surface has the beneficial effect of ventilating the lower atmosphere, which is typically plagued by stagnation during the winter. Additionally, the advection of dust from Region A is dependent on surface emission within the source region, which is reduced during winter because of increased soil moisture. Finally, the scenario of low-level westerlies in the winter is typical during the passage of a mid-latitude system, which may promote precipitation and vertical mixing and hence act as an aerosol sink.

### 4.4. Weekly Cycle of Visibility

**Figure 9:**
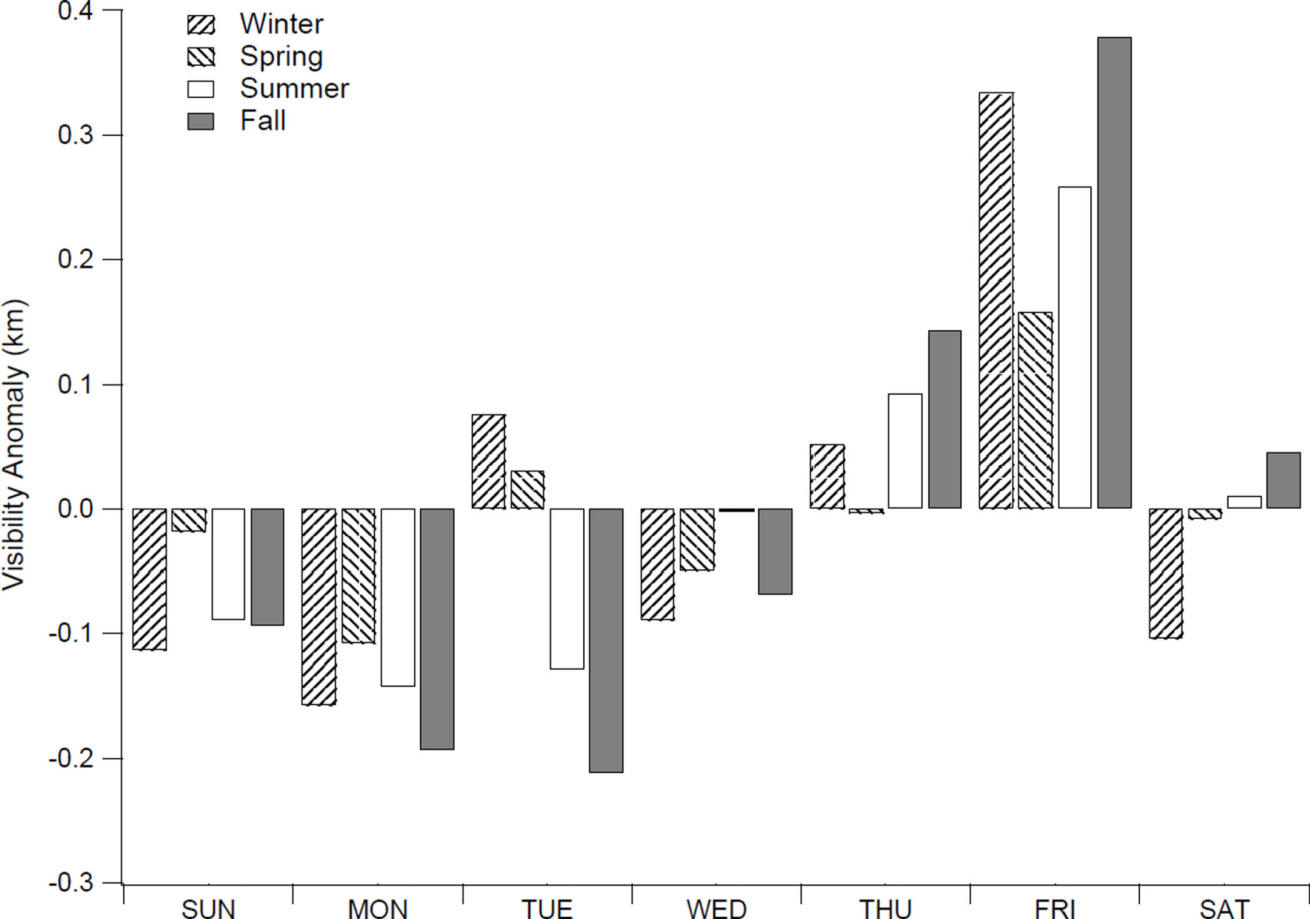
Average visibility anomaly in Tehran (Mehrabad) filtered by day-of-week for each season of the year. The visibility anomaly is calculated as the average deviation for each day-of-week from the climatological mean for each season. Note that the weekend in the study region is Friday, although some industries also observe Thursday as a reduced working day.

Another potentially important modulator of local aerosol concentrations is the weekly cycle of human activity, since anthropogenic emissions are expected to vary between workdays and weekends. It should be clarified that typically only Friday is the weekend in Iran; for some industries Thursday is also a reduced working day. Figure 9 shows the average visibility anomaly at Mehrabad for each day of the week. The anomaly is calculated as the average deviation from the seasonal mean visibility for each of the four seasons, which allows an independent comparison of the weekly cycle to be made without incorporating the significant seasonal variability in visibility. In all seasons, Friday exhibits a strong increase in visibility, which is aligned with an expected reduction in anthropogenic emissions. In addition, the visibility on Thursday is also anomalously high which would be supported by reduced working hours. With minor exceptions, the other days show broadly consistent visibilities. Another notable feature in these data is that the increased visibility on Friday is stronger in winter and fall compared with spring and summer. If the weekly cycle were used as an indicator of the relative importance of local (anthropogenic) sources compared to regional and meteorologically driven sources (e.g., dust), then this result would support the conclusion that local sources are more important in winter and fall for modulating aerosol concentrations. Conversely, during the spring and summer, regional dust transport, local dust sources and possibly biomass burning overshadow local anthropogenic emissions, which are strongly mixed in the deep summer mixing layer and so the apparent importance of the weekly cycle is reduced. The weekly cycle was also analyzed for the satellite AOD data (not shown) and no significant pattern emerged. This further supports the argument that urban sources play a secondary role to regional transport and meteorology in modulating the column aerosol properties, even though they are an important local influence for conditions at the surface.

## 5. Conclusions

This study has presented a multi-year aerosol characterization for metropolitan Tehran and surrounding areas using a combination of surface station data, satellite data, reanalysis data, and the GOCART model data. The scope of this paper was outlined in three questions posed earlier, and here we conclude by briefly responding to each in order:

- The local meteorology is shown to be strongly influenced by the presence of significant topography in the region and has a fundamental role in modulating the seasonal patterns of aerosol-related parameters. In winter, the mountains help trap air at low levels causing stagnant air to build near the surface resulting in poor visibility in the city. The reduced visibility is not as significant in areas outside the city and is suggestive of the influence of local emissions from the city. Even though visibility is poor during winter, the satellite-derived AOD values are at a minimum, which confirms that aerosols are trapped in the lowest layers and hence this promotes an increased sensitivity to local anthropogenic sources. The inverse seasonal variation of AOD and visibility is a major finding of this study. The satellite-derived AOD values increase during the late spring and summer due to a combination of local and regional dust sources and (perhaps) biomass burning.
- While precipitation is relatively low throughout the year, the summer is particularly dry and the lack of wet scavenging is partly responsible for the high AOD seen in the summer. During winter, processes associated with rain (e.g., wet scavenging, vertical mixing, and advection) appear to decrease AOD and provide a transient increase in visibility. The data show a consistent decrease in AOD and increase in surface visibility at Mehrabad during the two days after rain compared with the two days before rain.
- There is a positive visibility anomaly observed on Friday and, to a lesser extent on Thursday, which points to reductions in local emissions during the weekend. The size of the anomaly increases during winter and fall, which further strengthens the argument that low level aerosols are more sensitive to local anthropogenic sources during these seasons compared to spring and summer when dust transport, fires, and increased mixing overshadows local emissions.

## Figures and Tables

**Table 1:** Summary of temporal and spatial characteristics of datasets used.

Data Source	Data Type	Latitude (°)	Longitude (°)	Altitude (m AMSL)	Date Range
NCDC: Tehran Mehrabad	Met/Visibility	35.68	51.32	1191	1/1/2000–12/31/2009
NCDC: Semnan	Met/Visibility	35.55	53.38	1131	1/4/2000–12/31/2009
NCDC: Gharakhil	Met/Visibility	36.45	52.82	14	1/25/2000–12/31/2009
NCDC: Mehrabad	Radiosonde	35.68	51.32	1191	1/1/1980–12/31/2012

MODIS TERRA	Deep Blue AOD, Cloud Fraction	35–36	51–52	N/A	3/1/2000–12/31/2009
MODIS AQUA	Deep Blue AOD, Cloud Fraction	35–36	51–52	N/A	7/4/2002–12/31/2009
MODIS	FIRMS	20–55	20–70	N/A	3/1/2000–12/31/2009
MISR	AOD	35–36	51–52	N/A	2/25/2000–12/31/2009
TOMS	UV Aerosol Index	35–36	51.25–52.5	N/A	2/24/2000–12/14/2005
OMI	UV Aerosol Index	35–36	51–52	N/A	2/24/2000–12/14/2005

GOCART	Speciated AOD	34–36	50–52.5	N/A	2/24/2000–12/31/2007
HYSPLIT	Back-trajectories	35.7	51.42	500, 1000, 3000	1/1/2000–12/31/2009
MERRA	Gridded Reanalysis	35.5–36.5	51.33–52.67	N/A	1/1/2001–12/31/2009
